# Field-based assessment of inhibitory control in black-tailed gulls using a cylinder task

**DOI:** 10.1007/s10071-025-01997-4

**Published:** 2025-08-02

**Authors:** Kaho Minami, Yuichi Mizutani, Sota Inoue, Hibiki Sugiyama, Yusuke Goto, Akiko Shoji, Ken Yoda

**Affiliations:** 1 Graduate School of Environmental Studies, Nagoya University, Furo, Chikusa, Nagoya, 464-8601 Japan; 2 Organization for Research Initiatives and Development, Doshisha University, 1-3 Tatara Miyakodani, Kyotanabe, 610-0394 Japan; 3 Institute for Advanced Research, Nagoya University, Furo, Chikusa, Nagoya, 464-8601 Japan; 4 Graduate School of Information Science and Technology, The University of Osaka, Suita, 565-0871 Japan

**Keywords:** Cognition, Inhibition, Detour, Learning, Seabird, Laridae

## Abstract

One of the executive functions, inhibitory control, enables animals to suppress ineffective behaviors and facilitate flexible behavior. Seabirds, particularly those of the family Laridae, exploit diverse food resources across various environments. This suggests a possible link between their foraging behavior and inhibitory control. However, to date, inhibitory control in seabirds has not been assessed. We used a cylinder task to assess inhibitory control in wild black-tailed gulls, which are highly omnivorous seabirds. The task required gulls to suppress the dominant response of pecking at food inside a transparent cylinder, detour to the side openings, and retrieve the food without pecking the cylinder. The trial was considered successful if the gull retrieved the food without pecking the cylinder. Ten of the 12 individuals succeeded in the task within 10 trials, with their success rates improving across trials. These findings suggest that black-tailed gulls exhibit moderate levels of inhibitory control among birds and may learn detouring behavior through repetition.

**Supplementary Information:**

The online version contains supplementary material available at 10.1007/s10071-025-01997-4.

## Introduction

Inhibitory control, an executive function, plays a crucial role in facilitating both basic and complex cognitive functions, ranging from basic motor self-regulation—which involves suppressing ineffective responses to stimuli (Hauser 1999)—to higher-level self-control, which enables flexible behavior (Diamond 1990; MacLean et al. 2014). Inhibitory control may help animals suppress persistent foraging in one area and facilitate a flexible switch to more efficient foraging behavior (Coomes et al. 2022).

Inhibitory control has been studied in various bird species (MacLean et al. 2014; Kabadayi et al. 2016, 2017), with certain corvid species having exceptional abilities (MacLean et al. 2014; Kabadayi et al. 2016). This can potentially be linked to their dietary diversity because inhibitory control may contribute to suppressing dominant responses toward familiar foods in favor of exploring and exploiting new dietary resources (MacLean et al. 2014; Vernouillet et al. 2016). However, most prior research has focused on terrestrial birds, and to our knowledge, inhibitory control in seabirds has not been explored. Examining this trait in seabirds, with their unique evolution and ecology, deepens our understanding of their cognitive ecology and contributes to comparative ornithology.

The family Laridae (order Charadriiformes) provides a compelling model for investigating inhibitory control. Gulls are omnivorous, foraging across diverse environments from open oceans to human-altered habitats (Yoda et al. 2012; Enners et al. 2018). We hypothesized that gulls also exhibit high inhibitory control during task performance, given that their opportunistic foraging strategies resemble those of corvids.

In this study, we assessed the inhibitory control of wild black-tailed gulls (*Larus crassirostris*) using the cylinder task. Here, animals must detour around a transparent cylinder to access a visible food reward. The cylinder often triggers a reflexive response to reach directly for the food, causing them to bump into it (Diamond 1990). Inhibitory control suppresses this response by detouring to openings (Kabadayi et al. 2018). Using the cylinder task, we quantified gulls’ inhibitory control by measuring their success rate in retrieving food without pecking the cylinder. Since gulls rely on vision for foraging (Håstad et al. 2005), this visually driven task is expected to effectively assess inhibitory control in this species. We predicted that the gulls would exhibit a high success rate in retrieving food without pecking the cylinder, indicating a high level of inhibitory control.

## Methods

### Study site and species

We studied black-tailed gulls at a breeding colony on Kabushima Island, Japan (40°32′18″ N, 141°33′27″ E). The cylinder task was conducted from May 28 to June 5, 2024, during the chick-rearing period. Since males are larger than females in this species (Narita and Narita 2004), we determined the sex of each subject based on body size differences observed during incubation shifts. We marked the feathers of each subject with black dye for individual identification, varying the dyed body part (e.g. head, chest, or back), area, and pattern across individuals. The study involved 20 parent gulls from 17 nests, with both parents being tested in four nests. Subjects consisted of 11 females and nine males raising chicks aged 5–15 d. All of the trials were conducted when one parent stayed at the nest while the other was off-nest. None of these individuals had been previously tested in any inhibitory control task.

### Setup and materials

**Fig. 1:**
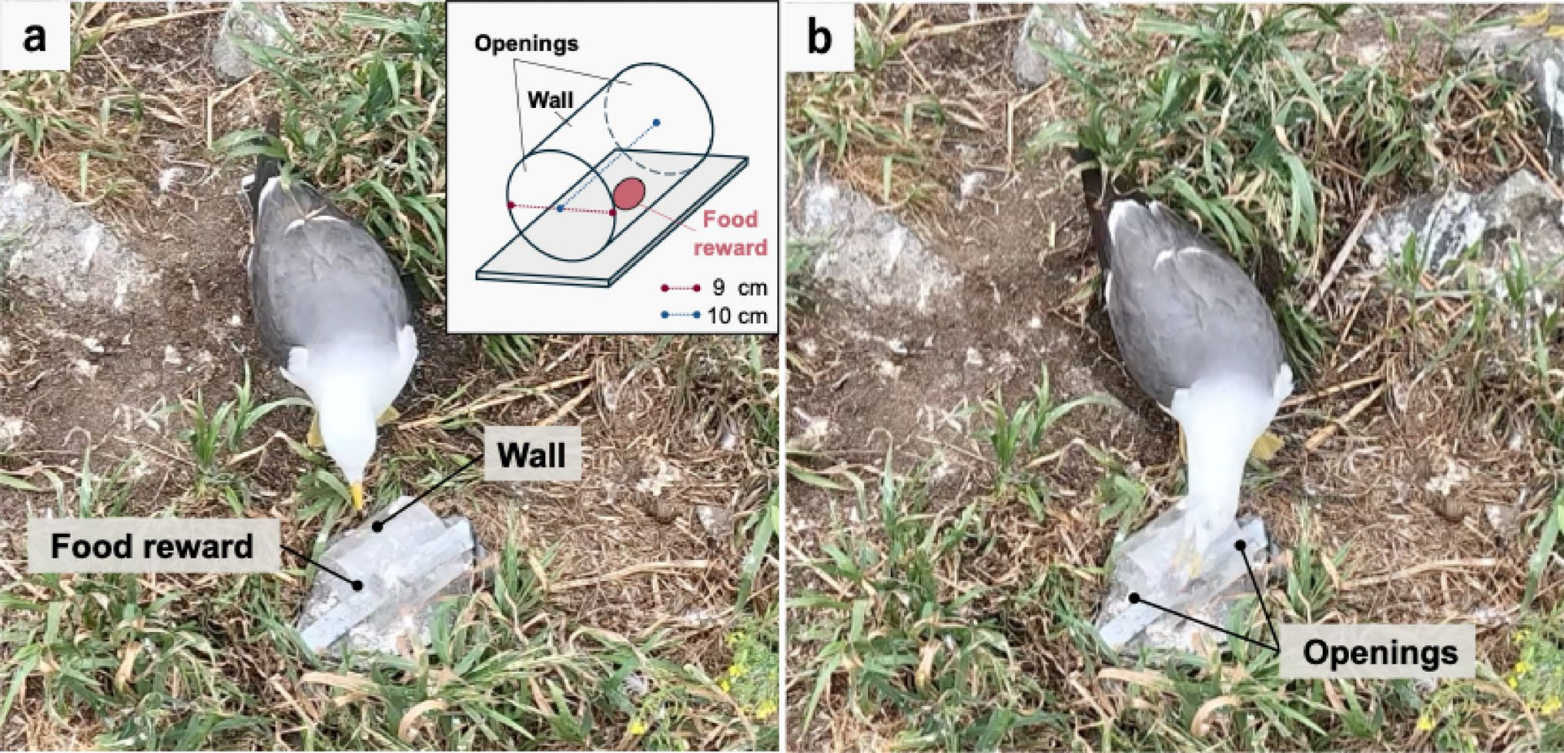
Overview of the cylinder task. (**a**) A trial was classified as a failure if the gull pecked the cylinder wall. (**b**) The gull retrieving food from the cylinder opening

We used a transparent cylinder, 10 cm in length and 9 cm in diameter, with openings on both ends, fixed on a flat stone (Fig. 1). As a food reward, we provided approximately 5 g of Japanese sardine (*Sardinops melanostictus*), a primary prey species for black-tailed gulls (Narita and Narita 2004). All trials were recorded using a GoPro HERO11 (GoPro, Inc., USA) mounted on a tripod positioned perpendicular to the cylinder wall, 1 m away from the apparatus.

### Procedure

We conducted two habituation phases before the experimental trials: Habituation 1 and Habituation 2. Following Ashton et al. (2018), we omitted the opaque condition, as prior exposure may induce rule learning that confounds performance in the transparent trial (Ashton et al. 2018).

In Habituation 1, the apparatus was placed near the target nest without a food reward, allowing subjects to familiarize themselves with it for at least 2 h. In Habituation 2, food was alternately placed at both sides of the cylinder openings, and subjects’ motivation and habituation were confirmed by whether they ate the food. Habituation 2 lasted up to 5 min per side, during which subjects that ate food from both sides proceeded to the trials, whereas individuals that did not meet this criterion were re-exposed to the procedure starting from Habituation 1. Subjects that still failed Habituation 2 were excluded from further trials.

Experimental trials began immediately after Habituation 2. The cylinder was aligned with its wall facing the direction from which the subject had approached during Habituation 2. The food was randomly placed by hand on either side on each trial, and both hands were simultaneously inserted into the cylinder to place the food at its center. The experimenter placed the food with their back turned to the subject to conceal the food placement during all trials. Each trial lasted 1 min, starting when the subject approached within 1 m of the apparatus. Each subject underwent 10 consecutive trials in a single day. The food was removed and replaced if the subject failed to retrieve it. During Habituation 2 and the trials, the experimenter approached the cylinder from the direction of the tripod and placed food while facing away from the gull. We also recorded the response time, defined as the duration between the gull approaching within 1 m of the apparatus and retrieving the food. The participation of a non-target individual occurred only once, in that case, we stopped the trial and did not count it as a valid trial.

### Statistical analysis

A successful trial was defined as the food being retrieved without the cylinder being pecked (Fig. 1). Subjects were classified as unmotivated if they neither pecked the apparatus nor retrieved the food within 1 min. Therefore, not all individuals completed 10 motivated trials. We calculated average success rates both including all trials regardless of subject motivation and excluding trials in which subjects were unmotivated.

We analyzed the success rate trend across motivated subject trials with a binomial generalized linear mixed model (GLMM) using the glmmTMB package (version 1.1.9, R version 4.3.2; Brooks et al. 2017). To clarify the effect of learning, we excluded individuals who succeeded on their first trial (i.e. those who succeeded without a learning phase) from this analysis. Trial number and sex were included as fixed effects, with success (1) and failure (0) as the response variable modeled using a logit link function. Random effects included individual ID as random slopes for trial numbers and random intercepts. To account for variation in the number of individuals participating in each trial, we included the log-transformed number of individuals per trial as an offset term. We applied a GLMM with gamma distribution and log link function to analyze the response times for food retrieval during trials where gulls successfully retrieved the food, regardless of whether they pecked the cylinder (see Supplementary Material). Fixed effects and random effects were the same as in the binomial GLMM. To assess whether the GLMMs had sufficient power to detect a sex effect, we conducted a simulation-based post-hoc power analysis. Using fixed effect estimates and random-effect variances from the fitted model, we simulated 1000 datasets assuming the observed sex effect was real. Each dataset was reanalyzed with the same model, and the p-value for sex was extracted. Power was defined as the proportion of simulations with *p* < 0.05. Additionally, to assess whether subjects may have learned by observing other subjects, we tested for a correlation between the order of trials and the number of trials to first success using Spearman’s rank correlation test, conducted both including all trials and excluding unmotivated subject trials.

## Results

Of the 20 individuals, 12 (four females and eight males) passed the habituation and proceeded to the trials. In the first trial, one individual was unmotivated, while the remaining 11 either pecked the cylinder or retrieved the food. Four succeeded, four retrieved the food after pecking, and three failed to retrieve the food within the time limit despite pecking the cylinder (Table 1).

**Table 1:** The results of the first trial for each individual. Retrieving the food without pecking was considered a success, while pecking was considered a failure, regardless of food retrieval

	Retrieved food	Did not retrieve food
Without peck	4	1^※^
With peck	4	3

^※^ unmotivated subject trial

**Fig. 2:**
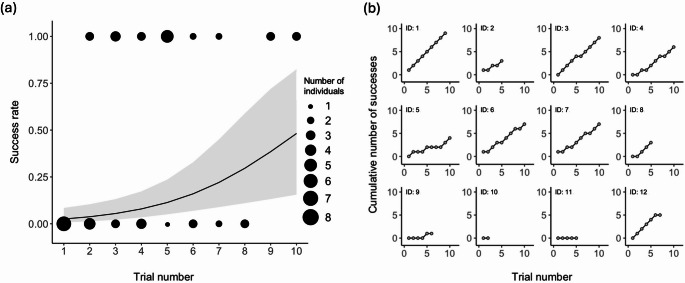
(**a**) Trend in the estimated success rate across motivated subject trials. The vertical and horizontal axes show the proportion of successes and trial number, respectively. Observed data points (1 = success, 0 = failure) are scaled by the number of subjects. The line indicates GLMM estimates, with the ribbon indicating the 95% confidence interval. The estimated success rate increased with trials (*p* = 0.0021). (**b**) Cumulative number of successes by individual in motivated subject trials. ID: 1, 2, 6, and 7 succeeded from the first trial and were therefore excluded from the analysis of success rate trends

Across all trials, 10 individuals succeeded at least once during the 10 trials. Of the 120 total trials, 89 were classified as motivated. The success rate was 43.3% (number of individuals = 12) across all trials and 58.4% (number of individuals = 12) for motivated subject trials. The mean number of trials to reach success was 2.40 (number of individuals = 10, range = 1–6, SD = ± 1.78), and for motivated trials, the mean was 2.10 (number of individuals = 10, range = 1–5, SD = ± 1.29; Table 2). Note that ID: 10 and 11, who did not succeed within 10 trials, were excluded from the mean calculations. All 12 individuals pecked the cylinder in at least one trial. However, none pecked the platform under the cylinder or the cylinder when it lacked food. The first interaction in each trial with the apparatus occurred after an average of 8.48 s (number of individuals = 12, number of trials = 89, range = 1–48, SD = ± 10.18), and in no trial did an individual interact with the apparatus at the moment of the time limit, and thus the experimenter never had to forcibly interrupt the interaction. The success rates increased across motivated trials (number of individuals = 8, *p* = 0.0021, z = 3.08; Table 3; Fig. 2). There was no significant difference in success rates between sexes (number of individuals = 8, *p* = 0.20, z = −1.27). However, the post-hoc power analysis revealed low statistical power (0.21%) to detect the sex effect, suggesting that the current GLMM may have lacked sufficient sensitivity to detect this effect. Spearman’s rank correlation tests revealed no significant relationship between trial order and the number of trials to first success, in both analyses including all trials (number of individuals = 10, ρ = 0.0063, *p* = 0.99, S = 163.95) and for motivated trials (number of individuals = 10, ρ = 0.00, *p* = 1.00, S = 165.00). Two individuals who did not succeed within 10 trials were excluded from this analysis.

**Table 2:** Summary of experimental data for the 12 black-tailed gulls that participated in the trials. The table shows the number of motivated trials, successful trials (defined as retrieving food within 1 min without pecking at the cylinder), and number of trials to success

ID	Sex	Number of motivated subject trials	Number of successful trials	Number of trials taken to reach success during all trials	Number of trials taken to reach success (during motivated subject trials)
1	M	9	9	1	1
2	F	5	3	1	1
3	F	10	7	2	2
4	F	10	6	3	3
5	M	10	4	2	2
6	M	10	7	1	1
7	F	10	7	1	1
8	M	5	3	5	3
9	M	6	1	6	5
10	M	2	0	No success	No success
11	M	5	0	No success	No success
12	M	7	5	2	2

**Table 3:** The results of a binomial GLMM analyzing changes in success rate across motivated subject trials

Fixed effects	Estimate	SE	z	*p*
(Intercept)	−3.11	0.89	−3.50	0.00046 ***
Trial number	0.40	0.13	3.08	0.0021 **
Sex (M)	−0.92	0.73	−1.27	0.20

**p < 0.01, ***p < 0.001

## Discussion

This study demonstrated that most of the black-tailed gulls succeeded in the cylinder task, highlighting its effectiveness for measuring inhibitory control in this species. The fact that 11 individuals interacted with the apparatus during the first trial suggests a minimal impact of neophobia (i.e. fear of novel objects or situations). The lack of pecking on the apparatus without food confirms that the observed pecking behavior was food-driven.

Ten of the 12 individuals succeeded at least once during the trials, with a success rate of 43.3% across all trials and 58.4% in motivated trials. Although direct comparison is difficult due to differences in experimental setups, these rates are lower than those observed in corvids (ravens *Corvus corax*: 100%, New Caledonian crows *Corvus moneduloides*: 92%, jackdaws *Corvus monedula*: 97%; Kabadayi et al. 2016). However, the gulls’ performance was comparable to that of some passerines, pigeons, and parrots (song sparrow *Melospiza melodia*: 27%, zebra finch *Taeniopygia guttata*: 52%, domestic pigeon *Columba livia*: 33%, orange-winged amazon *Amazona amazonica*: 51%; MacLean et al. 2014; African gray parrot *Psittacus erithacus*: 35%, Blue-headed macaw *Primolius couloni*: 36%; Kabadayi et al. 2017). Zucca et al. (2005) reported that young herring gulls (*Larus argentatus*) outperformed young quails (*Coturnix* sp.) and canaries (*Serinus canaria*) in a maze-like detour task, potentially reflecting the inhibitory control abilities of gulls. This is supported by the increasing success rate across trials, suggesting that the gulls learned to detour around the cylinder—a learning trend similar to that observed in parrots and Clark’s nutcrackers (*Nucifraga columbiana*) (Vernouillet et al. 2016; Kabadayi et al. 2017).

The fact that some individuals did not participate in the cylinder task represents a limitation of this study. Some non-participating individuals may have had low appetite during the trials, possibly due to factors such as having just returned from foraging. In addition, non-participation may reflect the high individual specialization observed in many gull species (e.g. Westerberg et al. 2019). Although black-tailed gulls are generalists at the species level, some individuals may specialize on particular prey types and therefore may not have recognized the Japanese sardine pieces used in the task as food. Additionally, we acknowledge that it was difficult to fully control opportunities for social learning in this densely packed colony. We placed target nests at least 5 m apart and found no significant association between trial order and trials to first success indicating little influence from learning by observing others, but we cannot completely rule it out. Such logistical constraints are inherent to field-based study.

In this study, we observed that success rates increased over repeated trials conducted at one-minute intervals within a single day, suggesting that inhibitory-control performance is modulated by rapid, experience-dependent learning. If the same individuals were retested after a longer interval (e.g. one month), we would hypothesize higher success rates on the first trial, reflecting retention of the detour rule. Conversely, a return to baseline performance would imply that the learned rule decays quickly and that task success depends more on innate inhibitory capacity than on long-term memory.

The gulls’ inhibitory control may be linked to their behavioral flexibility, enabling them to optimize foraging by utilizing a broad range of food resources, including not only natural prey at sea but also fisheries discards at fishing ports and markets, and insects in rice fields (Yoda et al. 2012). Comparative studies across populations or species are needed to understand the relationship between inhibitory control and foraging behavior. The successful application of the cylinder task with black-tailed gulls highlights its potential for studying other gull species and ground-foraging Charadriiformes, offering valuable insights into seabird cognitive ecology.

## Supplementary Information

Below is the link to the electronic supplementary material.

Supplementary Material 1

## Data Availability

Data is provided within the manuscript and supplementary information files.
