# Supplementary material for: Field-based assessment of inhibitory control in black-tailed gulls using a cylinder task
Source: Anim Cogn. 2025 Aug 2;28(1):77. doi: 10.1007/s10071-025-01997-4 (PMC12317854; doi:10.1007/s10071-025-01997-4)
Supplement: Supplementary file 1 — Supplementary Material 1 [file 10071_2025_1997_MOESM1_ESM.pdf]

Supplementary Material

Field-based assessment of inhibitory control in black-tailed gulls using a cylinder task

Journal Name: *Animal Cognition*

Authors' information

Kaho Minami[1], Yuichi Mizutani[2], Sota Inoue[1,3,4], Hibiki Sugiyama[1], Yusuke Goto[1], Akiko Shoji[1], Ken Yoda[1]

1: Graduate School of Environmental Studies, Nagoya University, Furo, Chikusa, Nagoya, Japan 464-8601

2: Organization for Research Initiatives and Development, Doshisha University. 1-3 Tatara Miyakodani, Kyotanabe, Japan 610-0394

3: Institute for Advanced Research, Nagoya University, Furo, Chikusa, Nagoya, Japan 464-8601

4: Graduate School of Information Science and Technology, The University of Osaka, Suita, Japan 565-0871

Corresponding author

Kaho Minami

E-mail: minami.kaho.k5@s.mail.nagoya-u.ac.jp

ORCID: 0009-0006-2926-1900

Methods

Statistical analysis

We analyzed the response times for retrieving the food in trials where the gulls retrieved the food, regardless of whether they pecked at the cylinder. We used the same GLMM as the success rate analysis, with the same fixed effects, random intercepts, random slopes, and offset term. The response variable was the time in seconds taken to retrieve the food and we applied a gamma-distributed GLMM with a log link function. To assess whether this gamma GLMM had sufficient power to detect a sex effect, we conducted a simulation-based post-hoc power analysis. Using fixed effect estimates and random-effect variances from the fitted model, we simulated 1000 datasets assuming the observed sex effect was real. Response variables were generated from a gamma distribution with a log link using the estimated mean and a fixed shape parameter. Each dataset was reanalyzed with the same model, and the p-value for sex was extracted. Power was defined as the proportion of simulations with $p < 0.05$.

Results

The time to retrieve the food did not decrease regardless of pecking ($p = 0.45$; Table S1). The average response time was 10.6 s per trial (range: 2–58, SD ± 12.2). The sex effect on success rates was not significant ($p = 0.58$). However, the post-hoc power analysis revealed low statistical power (36.06%) to detect the sex effect, suggesting that current GLMM may have lacked sufficient sensitivity to detect this effect.

Table S1 The results of a gamma GLMM analyzing the response times in retrieving the food across the 72 trials, in which the food was retrieved regardless of pecking. The response times to retrieve the food did not decrease

Fixed Effects	Estimate	SE	z	p
(Intercept)	−0.21	0.41	−0.54	0.60
Trial number	0.076	0.057	1.34	0.18
Sex (M)	0.23	0.40	0.59	0.58
